# Acute primary repair of extraarticular ligaments and staged surgery in multiple ligament knee injuries

**DOI:** 10.1186/s10195-020-00557-5

**Published:** 2020-10-07

**Authors:** Yasuyuki Ishibashi, Yuka Kimura, Eiji Sasaki, Shizuka Sasaki, Yuji Yamamoto, Eiichi Tsuda

**Affiliations:** 1 Department of Orthopaedic Surgery, Hirosaki University Graduate School of Medicine, 5 Zaifu-cho, Hirosaki, Aomori 036-8562 Japan; 2 Department of Rehabilitation Medicine, Hirosaki University Graduate School of Medicine, 5 Zaifu-cho, Hirosaki, Aomori 036-8562 Japan

**Keywords:** Knee dislocations, Multiligament knee injuries, Primary repair, Staged surgery

## Abstract

**Background:**

The purpose of this study is to compare the outcomes of acute primary repair of extraarticular ligaments with staged surgery for acute knee dislocations (KDs) and multiligament knee injuries (MLKIs).

**Materials and methods:**

Between January 2005 and May 2018, 61 consecutive patients diagnosed with MLKI or KD were referred to or visited our institution. Of these, 31 patients who underwent acute repair of extraarticular ligaments within 3 weeks of injury were included in this study. These patients were retrospectively classified into two groups: those who underwent only primary repair (repair group) and those who underwent staged reconstructive surgery (staged group). Follow-up examination included range of motion (ROM), knee joint stability (Lachman test, posterior drawer test, and varus and valgus stress test), Lysholm knee score, Tegner activity scale, and Knee Injury and Osteoarthritis and Outcome Score (KOOS).

**Results:**

Twelve of the 31 patients did not need or desire further surgery and were included in the repair group. No significant difference was observed in demographic data between the repair and staged groups. Although staged surgery decreased positive posterior drawer test results, no significant difference was observed between the two groups regarding ROM, other knee joint stability tests, Lysholm scores, Tegner scale, or KOOS.

**Conclusions:**

In this series, all patients returned to their activities of daily living and preinjury occupation levels. Acute primary repair of extraarticular ligaments provides essential knee stability without varus/valgus instability and may reduce the need for subsequent cruciate ligament reconstruction.

**Level of evidence:**

Level IV, retrospective observational study.

## Introduction

Knee dislocations (KDs) and multiligament knee injuries (MLKIs) are severe knee traumas which involve intra- and extraarticular ligament tears, often with concomitant vascular and nerve damage and a fracture around the knee. Because popliteal artery lesion is a limb-threatening injury, early revascularization should be prioritized to avoid limb amputation [1, 2]. Concomitant other organ traumas, such as open fracture and head trauma, may similarly compromise the optimal timing of MLKI treatment. Therefore, it is difficult to apply a single approach and ideal surgical timing. Since MLKIs and KDs are uncommon and often heterogeneous, as mentioned above, minimal evidence is available, resulting in a lack of consensus regarding the most effective treatment [3, 4]. Although conservative and surgical treatments have been reported, surgical interventions have generally been recommended because of poor outcomes after conservative treatment [5–7]. Currently, conservative treatment is exclusively selected for patients who are unfit for surgery, frail, or sedentary [7].

Surgical intervention varies from the primary repair of damaged ligaments to anatomical ligament reconstruction in either a simultaneous or staged fashion [5, 8]. Early surgical treatment has been advocated to improve results [9–11], and the critical time to reestablish anatomic relationships is the first 3 weeks after injury [12]. Acute ligament reconstruction improves postoperative knee stability [13] and may increase the rate of arthrofibrosis, which causes deterioration in knee function and requires additional surgeries [14].On the contrary, delayed reconstruction may provide the time for natural healing of extraarticular ligaments and decrease postoperative arthrofibrosis [5, 15, 16]; it requires multiple grafts and tunnels for reconstructions, resulting in donor-site morbidity and risk of tunnel convergence [17]. Staged surgery, which involves repair of the extraarticular ligaments in the acute stage and subsequent reconstruction of the cruciate ligaments at a later stage, showed excellent clinical results [18]. However, staged surgery requires multiple surgeries, and this prolongs rehabilitation. Recently, the primary repair of knee ligaments, including the intraarticular ligaments, has attracted interest because it has the advantage of preserving the native tissues and avoiding the need for graft harvesting or more invasive surgery [19–21].

In our experience of MLKI treatments, some patients did not undergo proper primary repair because of polytrauma, requiring prolonged intensive care or revascularization surgery for popliteal arterial injury, and had significant residual knee instability despite undergoing delayed reconstruction. After experiencing these cases, we changed our surgical strategy to early repair of the extraarticular ligaments, especially the posterior capsule structure, at the time of revascularization surgery. Intraarticular cruciate ligament reconstructions are performed if the patient desires further surgery. The purpose of this study is to compare the outcomes of acute primary repair of extraarticular ligaments with staged surgery in acute KDs and MLKIs. We hypothesize that optimal primary repair of extraarticular ligaments not only improves the results of intraarticular cruciate ligament reconstruction but also reduces the frequency of cruciate ligament reconstruction.

## Patients and methods

### Patients

**Fig. 1:**
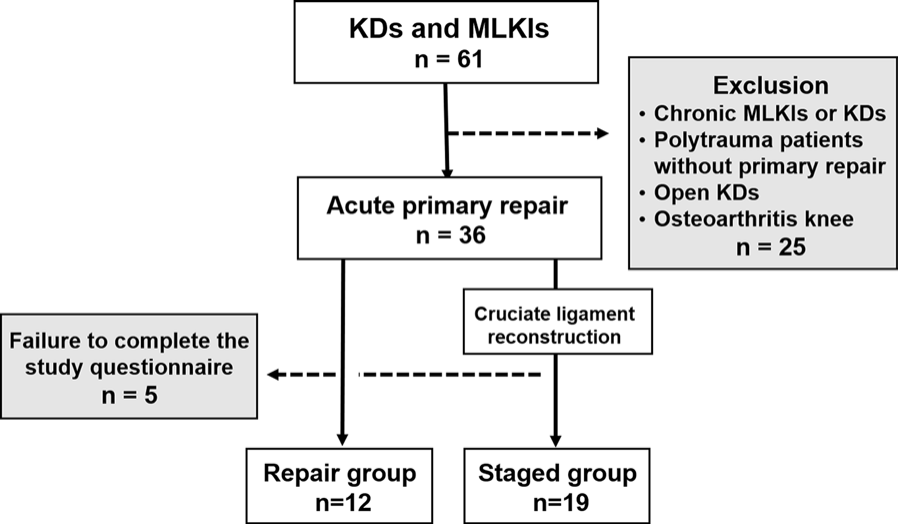
Flowchart of study. KDs: knee dislocations; MLKIs: multiligament knee injuries

In this study, MLKIs were defined as disruption of at least two of the four major knee ligament structures [anterior cruciate ligament (ACL), posterior cruciate ligament (PCL), posteromedial corner (PMC), and posterolateral corner (PLC)] [5]. Between January 2005 and May 2018, a consecutive series of 61 patients diagnosed with MLKI or KD were referred to or visited our institution. Inclusion criteria were (1) radiographically documented KD; (2) PCL injury with associated injuries to the PMC, including the medial collateral ligament (MCL), and/or associated injuries to PLC, including the lateral collateral ligament (LCL); (3) bicruciate ligament injury and associated injury to at least one collateral ligament (KD-IIIM or KD-IIIL) [22]; and (4) injury to all four major ligaments (KD-IV). On the contrary, exclusion criteria were (1) chronic MLKIs or KDs, (2) no acute primary repair because of prolonged intensive care, (3) open knee dislocation, (4) knees with osteoarthritis, (5) patients with ACL injury and grade III MCL injury who underwent simultaneous ACL reconstruction and MCL repair, and (6) failure to complete the study questionnaire (Fig. 1). Thirty-one patients who underwent acute primary repair of extraarticular ligaments within 3 weeks after injury met the inclusion criteria and formed the study group (Table 1). The average age at injury was 48.6 ± 21.3 years (14–80 years), and there were 21 men and 10 women. The mechanisms of injury were 15 high-energy traumas, such as traffic accidents and falls from heights greater than 2 m, 10 sports-related injuries, and 6 other low-energy traumas. These patients were classified into two groups: those who underwent only primary repair (repair group) and those who underwent staged surgery (staged group). After approval from our institution’s ethics committee, all patients provided informed written consent before inclusion in the study.

**Table 1:** Patient demographics

	All injured knees (*N* = 31)	Repair group (*N* = 12)	Staged group (*N* = 19)	*p*-Value
Age (years): mean ± SD (range)	48.0 ± 20.6 (14–75)	55.9 ± 17.5 (18–75)	42.9 ± 21.2 (14–73)	0.093
Sex (male:female)	21 (67.7%):10 (32.3%)	9 (75.0%):3 (25.0%)	12 (63.2%):7 (36.8%)	0.697
BMI (kg/m^2^): mean ± SD (range)	25.1 ± 4.4 (19.7–39.5)	25.7 ± 5.3 (20.8–39.5)	24.8 ± 3.8 (19.7–32.0)	0.703
Time to primary repair (days)	5.9 ± 5.5 (0–20)	6.3 ± 7.1 (0–20)	5.7 ± 4.5 (0–14)	0.646
Damaged ligaments *N* (%)				
PCL, PMC, and/or PLC	6 (19.3%)	3 (25.0%)	3 (15.8%)	0.879
ACL, PCL, MCL (KD III-M)	18 (58.1%)	7 (58.3%)	11 (57.9%)	
ACL, PCL, LCL (KD III-L)	3 (9.7%)	1 (8.3%)	2 (10.5%)	
ACL, PCL, MCL, LCL (KD IV)	4 (12.9%)	1 (8.3%)	3 (15.8%)	
Associated injuries, *N* (%)				
Nerve injury	3 (9.7%)	1 (8.3%)	2 (10.5%)	1.000
Vascular injury	5 (16.1%)	3 (25.0%)	2 (10.5%)	0.350
Follow-up (months) mean ± SD (range)	60.9 ± 31.7 (24–160)	50.0 ± 23.0 (24–78)	67.6 ± 34.9 (24–160)	0.164

*SD* standard deviation, *N* number, *BMI* body mass index, *ACL* anterior cruciate ligament, *PCL* posterior cruciate ligament, *PMC* posteromedial corner, *PLC* posterolateral corner, *MCL* medial collateral ligament, *LCL* lateral collateral ligament, *KD* knee dislocation

### Preoperative examination

**Fig. 2:**
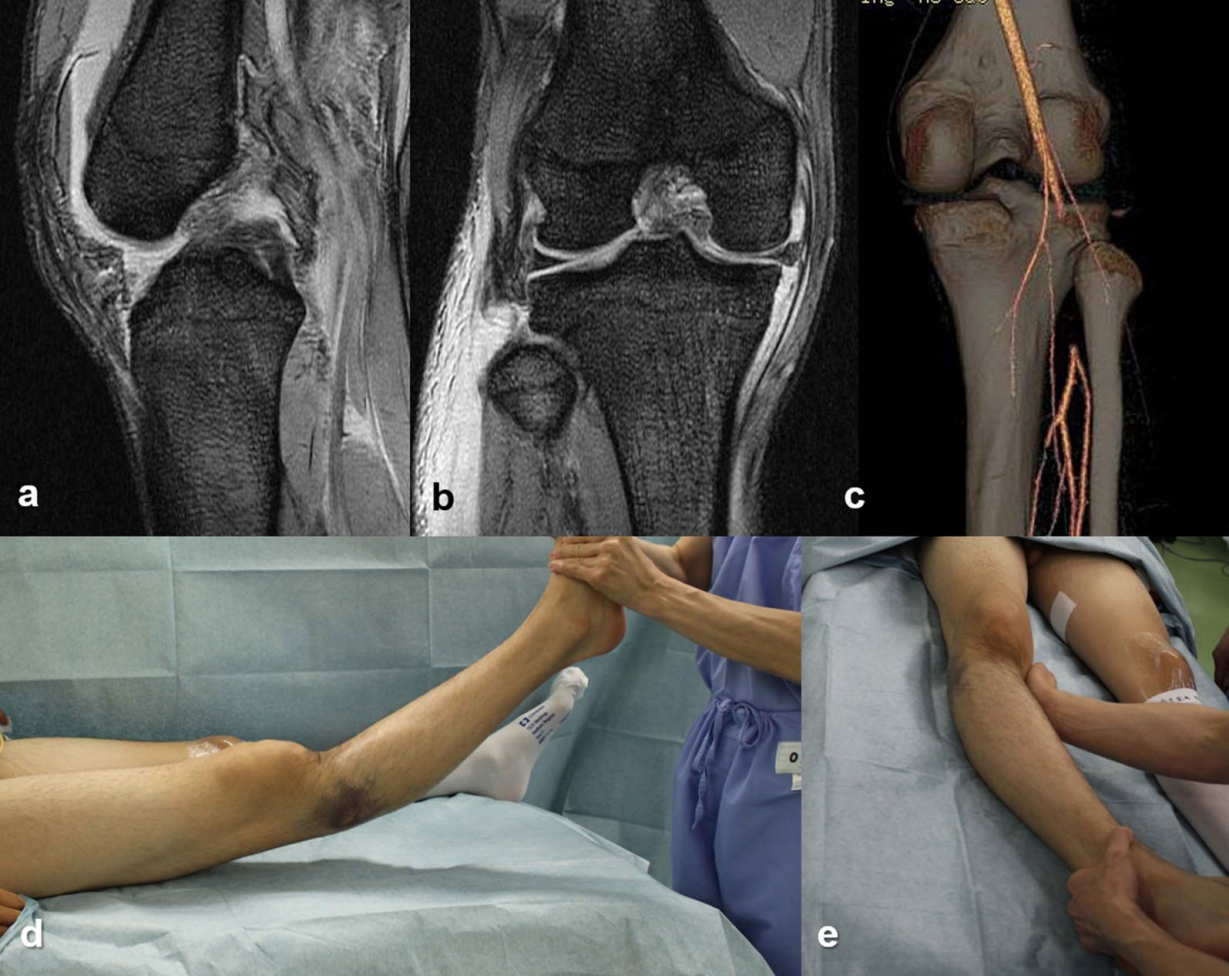
MRI and CT angiography and evaluation under anesthesia. An 18-year-old baseball player who suffered multiligament knee injuries during a baseball game. **a** Sagittal MRI showing PCL injury and posterior capsular injury; **b** coronal MRI revealing avulsion of the posterolateral complex from the fibular head; **c** angio-CT revealing occlusion of the popliteal artery; **d**, **e** evaluation under anesthesia showing severe posterior and varus instability

After administration, radiographic evaluations, including magnetic resonance imaging (MRI), were performed as soon as possible to determine surgical strategies (Fig. 2). Computed tomography (CT) angiography was always performed if the patient showed any suspected signs of popliteal arterial injury, such as ankle--brachial pressure index > 0.9. After examination of knee instability under anesthesia, diagnostic arthroscopy was quickly performed to assess associated intraarticular lesions in all patients except for those with vascular injury. Meniscal lesions, such as locked meniscus, were treated arthroscopically if observed.

### Acute primary repair

Patients were placed in supine position, and extraarticular medial-sided and lateral-sided injuries were repaired. Through medial or lateral longitudinal incision, damaged structures were carefully identified. The injured collateral ligament was sutured using pull-out sutures (no. 2 Ethibond; Ethicon, Somerville, NJ) from the intact attachment towards the avulsed ends. Subsequently, the avulsed ends were fixed to their anatomical insertion using soft suture anchors underpulling the Ethibond sutures. Similarly, the pull-out sutures were used to reinforce the fixation of the MCL or LCL to the surrounding soft tissues. The structures of the PMC and PLC were also anatomically fixed to their anatomical site of insertion using soft suture anchors. All injuries of the capsule around the joint were treated with primary repair using absorbable sutures (2-0 Vicryl; Ethicon) and smaller suture anchors to provide fixation points. If there were any avulsion fractures continuous with the ligaments, the fragment was fixed by screws. Since posterior structures of the knee become taut in an extension position, repair of these structures was completed with the knee held in extension. After primary repair, we confirmed whether the knee could be fully extended.

**Fig. 3:**
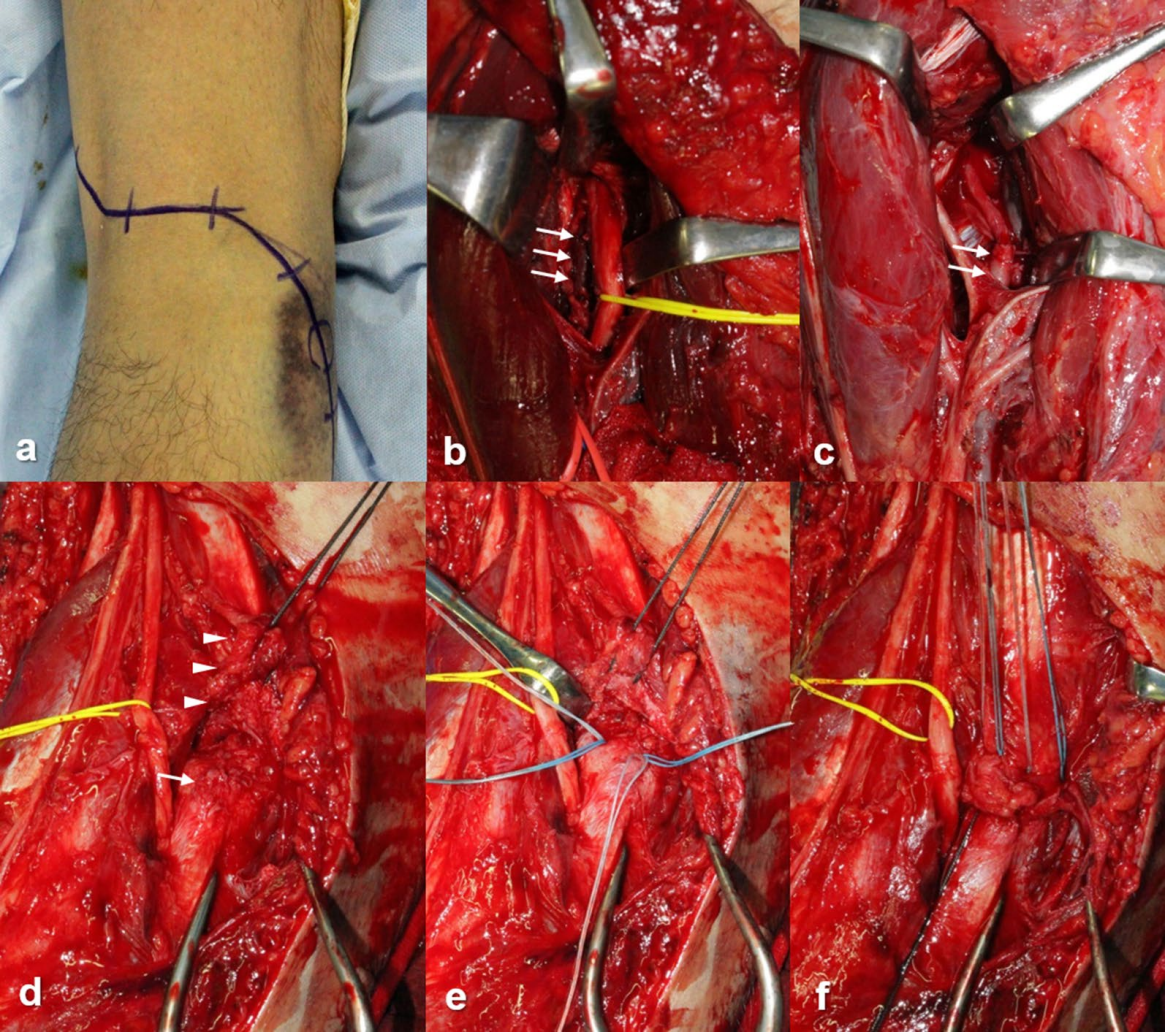
Vascular surgery and primary repair of posterolateral complex of left knee (same patient as in Fig. 2). **a** Skin incision for posterior approach; **b** popliteal artery thrombosed due to intimal rupture (arrows); **c** reversed saphenous vein graft (arrows), **d** PLC (arrow heads) avulsed from fibular head (arrow); **e** suture anchors inserted into fibular head; and **f** PLC fixed by suture anchors and torn posterior capsule repaired

**Fig. 4:**
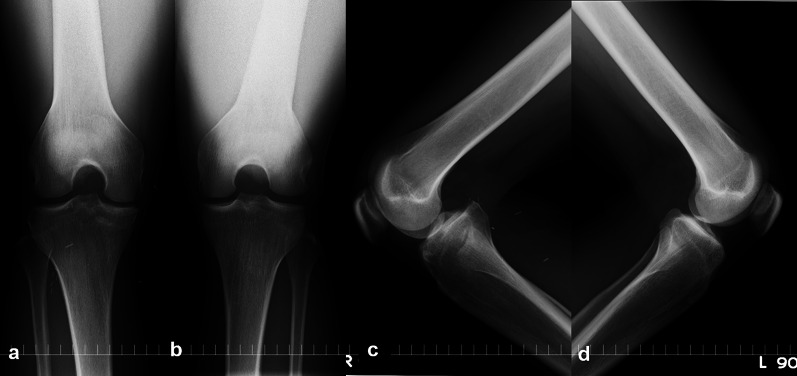
Postoperative radiograph (same patient as in Fig. 2). **a**, **b** Radiograph taken 4 years after primary repair showing no significant change in osteoarthritis; **c**, **d** posterior sag view of bilateral knee. Radiograph of right knee shows posterior laxity of 13 mm. This patient does not need further surgery and has returned to recreational baseball play without restriction

If the patient suffered popliteal arterial injury, emergent vascular surgery was primarily performed to prevent limb amputation. At our institution, orthopedic hand surgeons performed these vascular reconstructions, including primary arterial sutures and a reverse saphenous vein graft. Patients were placed in prone position, and their knees were slightly flexed (Figs. 3, 4). Through the posterior crank skin incision, vascular surgery was performed; and subsequently, acute primary repairs of extraarticular ligaments were performed through the same incision. For patients with peroneal nerve palsy, nerve release was performed, followed by primary repairs of extraarticular ligaments.

### Postoperative rehabilitation after primary repair

Postoperatively, the patient’s knee was fixed externally with a brace. Patients began isometric muscle-strengthening exercises, such as patella setting and straight leg raising, the day after surgery. If possible, patients were allowed non-weight-bearing gait with crutches as soon as possible. Range of motion (ROM) exercises using continuous passive motion devices and partial weight-bearing gait was commenced at the beginning of the third postoperative week. Patients progressed to full ROM exercise and full weight-bearing gait after 6 weeks. No open chain exercises were allowed for the first 3 months.

### Staged reconstruction

**Fig. 5:**
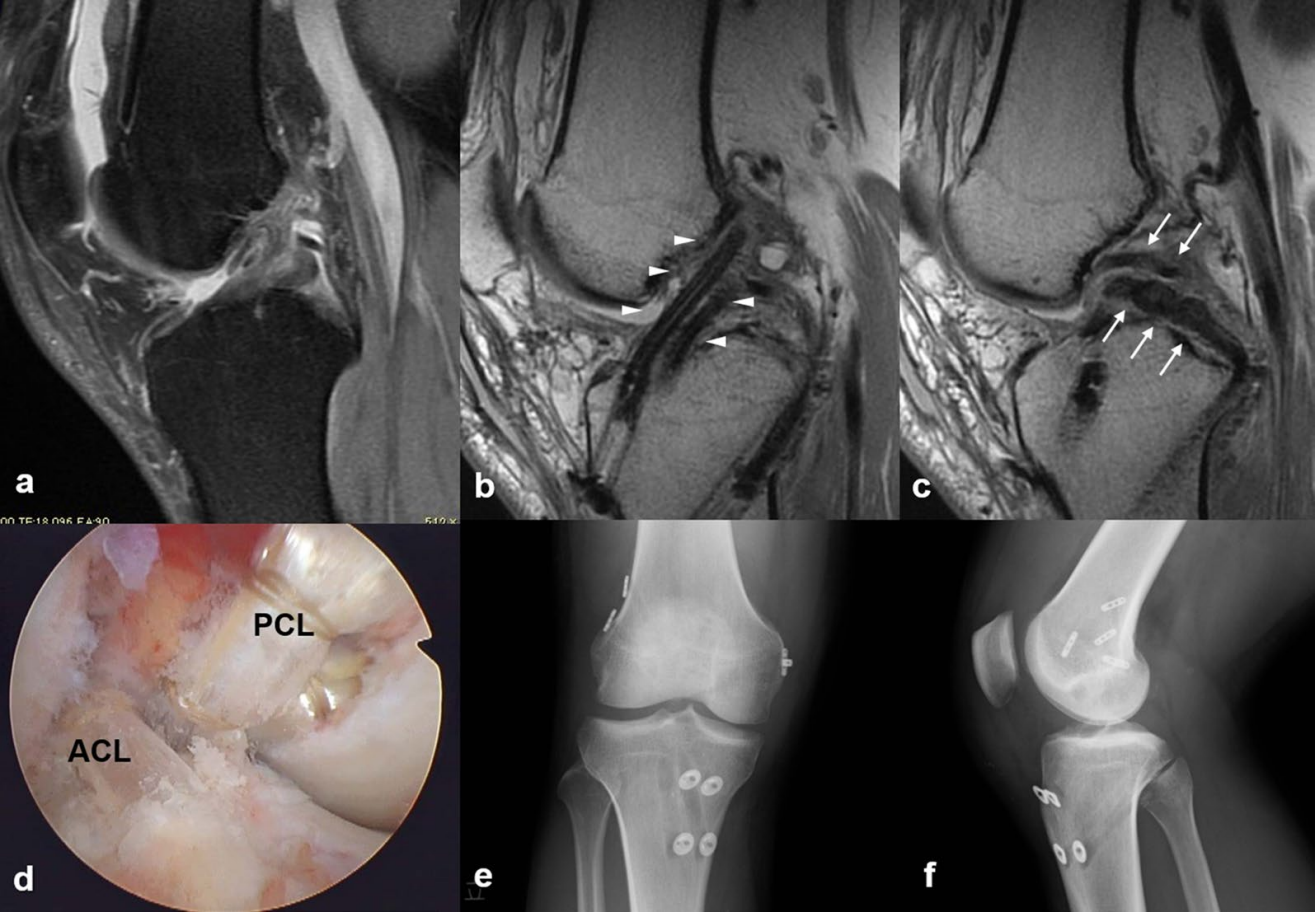
Double-bundle ACL and PCL reconstructions of right knee (26-year-old male judoist). **a** preoperative MRI showing torn ACL and PCL; **b**, **c** postoperative MRI showing reconstructed ACL (arrow heads) and PCL (arrows), **d** arthroscopic view of double-bundle ACL and PCL reconstructions, and **e**, **f** postoperative radiograph. ACL: anterior cruciate ligament; PCL: posterior cruciate ligament; MRI: magnetic resonance imaging

Cruciate ligament reconstruction was usually recommended for young, active patients as an elective surgery after primary repair. Once the patient had gained a sufficient ROM in their knees, they underwent staged surgery, usually approximately 6 months after primary repair. Cruciate ligament reconstruction was performed using a double-bundle technique with an ipsilateral autogenous hamstring tendon (Fig. 5). When simultaneous double-bundle ACL and PCL reconstructions were performed, contralateral hamstring tendons were also harvested. If the patient did not desire further surgery, the surgical treatment was completed exclusively with acute primary repair. After staged surgery, practically similar rehabilitation as after the primary repair was performed.

### Outcome assessments

Postoperative complications, such as infection, were assessed. Postoperative ROM and knee stability were assessed at final follow-up. Knee stability at final follow-up was defined by a Lachman test ≤ grade I, a posterior drawer test ≤ grade II, and varus and valgus instability ≤ grade II. The Lysholm knee score was used for assessment of daily functional activity at final follow-up, and activity levels at preinjury and final follow-up were evaluated with the Tegner activity scale. Subjective satisfaction was assessed using the Knee Injury and Osteoarthritis and Outcome Score (KOOS) [23]. These results were compared between the repair and staged groups.

### Statistical analysis

Descriptive data are presented as mean ± standard deviation for continuous variables, and the Tegner activity scale was calculated with median and range values. Preoperative demographics, postoperative ROM, and clinical scores, including the Tegner activity scale, the Lysholm score, and the KOOS, were compared between the repair and staged groups using the Mann–Whitney *U* test. In addition, type of knee dislocation, rate of concomitant peroneal nerve or vascular injury, and presence of knee instability were compared between the two groups using a chi-square test or Fisher’s exact test. Data input and analyses were performed using SPSS version 25.0 J (SPSS Inc., Chicago, IL). *p*-Value < 0.05 was considered statistically significant.

## Results

Twelve of the 31 patients did not want or need further surgery and were included in the repair group (38.7%). The other 19 patients underwent cruciate ligament reconstruction and were included in the staged group (61.3%). The average age in the repair group was 55.9 ± 17.5 (18–75) years, and there were nine men and three women. The average age in the staged group was 42.9 ± 21.2 (14–73) years, and there were 12 men and 7 women. The follow-up periods in the repair and staged groups were 50.0 ± 23.0 (24–78) months and 67.6 ± 34.9 (24–160) months, respectively. No significant difference was observed in patient characteristics between the repair and staged groups (Table 1). The most frequent injury was a combination of ACL, PCL, and MCL (KD III-M) occurring with a frequency of 58.1%.

No significant complications were observed after either primary repair or staged surgery. One patient had skin necrosis requiring free skin grafting after popliteal arterial surgery in the primary repair group. Regarding clinical outcomes at final follow-up, no significant difference was observed between the two groups regarding ROM or knee stability, except posterior instability (*p* = 0.006) (Table 2). No patients presented postoperative grade III varus or valgus instability in either group. Postoperative Tegner activity scales decreased compared with preinjury ones in both groups, and all patients returned to their activities of daily living and preinjury occupational level. Lysholm score, Tegner activity scale, and all subscales of the KOOS did not differ between the two groups.

**Table 2:** Postoperative range of motion, knee stability, and outcome score in repair and staged groups

	Repair group (*N* = 12)	Staged group (*N* = 19)	*p*-Value
ROM			
Extension (°): mean ± SD (range)	−2.9 ± 3.2 (−10–0)	−1.4 ± 3.3 (−10–0)	0.104
Flexion (°): mean ± SD (range)	132.5 ± 16.3 (110–150)	134.4 ± 11.7 (105–150)	0.950
Knee stability			
Positive Lachman (≤ grade 2): *N* (%)	3 (25.0%)	1 (5.3%)	0.279
Positive posterior drawer (≤ grade 2)	9 (75.0%)	2 (10.5%)	0.006
Varus instability (grade 2): *N* (%)	1(8.3%)	1 (5.3%)	1.000
Valgus instability (grade 2): *N* (%)	1 (8.3%)	0 (0%)	0.387
Clinical score			
Preinjury Tegner score: mean (range)	4.1 (1–7)	4.7 (1–8)	0.346
Postop Tegner scale: mean (range)	3.3 (1–6)	4.0 (1–8)	0.491
Lysholm score: mean ± SD (range)	87.4 ± 18.5 (44–100)	84.9 ± 19.1 (39–100)	0.537
KOOS: mean ± SD (range)			
Pain	77.3 ± 21.0 (36.1–100)	74.8 ± 21.5 (36.1–100)	0.827
Symptom	75.6 ± 20.6 (42.9–100)	70.2 ± 17.2 (39.3–92.9)	0.610
ADL	79.0 ± 16.3 (57.4–100)	83.5 ± 23.0 (35.3–100)	0.294
Sport/rec	54.5 ± 36.6 (0–100)	57.0 ± 34.8 (10–100)	0.680
QOL	59.7 ± 28.3 (25–100)	61.3 ± 30.1 (25–100)	0.680

*SD* standard deviation, *N* number, *KOOS* Knee Injury and Osteoarthritis and Outcome Score, *ROM* range of motion, *ADL* activities of daily living, *QOL* quality of life

## Discussion

The most important finding of this study is that acute primary repair of the extraarticular ligament provided satisfactory results for both KDs and MLKIs, and practically similar results were obtained for the staged surgery without any adverse effects. In this series, 12 of 31 patients (38.7%) did not need second cruciate ligament reconstruction and were satisfied with their results. Bin and Nam assessed the results of the two-stage management of their MLKI patients and similarly reported that one-third of patients did not require second-stage surgery [18]. They concluded that second-stage surgery was only performed in cases where it was deemed necessary. Based on these results, extraarticular ligament repair may ensure minimal essential knee stability.

Several authors have reported systematic reviews of staged surgery for MLKIs [6, 24]. Based on surgical timing, Mook et al. [24] and Jian et al. [6] classified MLKI treatments into three groups: acute (ligamentous surgery performed less than 3 weeks after injury), chronic (ligamentous surgery performed more than 3 weeks after injury), and staged (both acute and chronic surgery). They concluded that staged surgery yielded the best clinical results for MLKIs, although no significant difference was observed between the acute and chronic surgery groups in clinical outcomes. Similarly, Mook et al. demonstrated that patients who were managed acutely had more flexion deficits than those who were managed chronically. They suggested that more aggressive rehabilitation might prevent ROM deficits from occurring in acutely treated MLKIs. In other systematic reviews comparing early versus late surgical treatment of MLKIs [5, 25], early surgical treatment showed a significantly superior clinical outcome compared with late reconstruction. Hohmann et al. reported that total ROM did not significantly differ between the two groups.

The MCL and PMC, including the posterior oblique ligament, are the most commonly injured structures in MLKIs [26]. The PMC controls valgus and internal rotation as well as posterior drawer in extension [27]. Therefore, the PMC should be treated appropriately with the damaged MCL. The MCL and PMC can be treated with either primary repair or reconstruction [7]. Since the quality of the damaged medial structures is usually robust enough to facilitate a satisfactory repair [26], these structures should be repaired during the acute phase. A systematic review of medial knee ligament injuries demonstrated that repair of the MCL and PMC was an effective and reliable treatment [28]. Primary repair of these structures improved not only valgus stability but also patient-reported functional scores with low rates of secondary failure. An acute primary repair can also preserve grafts for later staged surgery of the cruciate ligament.

The PLC is important to control varus and rotational stability of the knee, and PLC injuries have a higher incidence than previously reported [29]. Since Stannard et al. demonstrated that results with repair followed by early motion rehabilitation were significantly inferior compared with results from reconstruction [30], PLC reconstruction has become a more popular procedure than primary repair. Similarly, Levy et al. recommended reconstruction of the PLC structures based on their comparative cohort study [31]. PLC injury sometimes involves femoral peel-off lesions that can be successfully managed with primary repair [32]. In this series, repair of the PLC structure demonstrated satisfactory results, apparently because they were all repaired in the acute phase, and recent suture anchors could be used.

Supposedly, the key factor for successful treatment of MLKIs is to maintain a proper positional relationship between the femur and tibia in knee extension shortly after injury. Since posterior structures, including the PMC and PLC, become taut with knee extension, these structures have a critical role in stabilizing the knee in an extension position. Furthermore, the posterior capsule and oblique popliteal ligament (OPL) are quite strong structures that contribute to the stability of knee extension [33]. Therefore, these structures should be equally repaired. However, current surgical procedures usually ignore the repair of the posterior capsule or the OPL despite their being vulnerable structures in MLKIs.

One study reported that popliteal artery injury associated with MLKI significantly decreased knee function scores compared with those without vascular involvement [34]. However, most studies on MLKIs exclude patients with popliteal artery injury, and their clinical outcomes remain unknown. In this series, five cases had popliteal artery injury and were treated with vascular anastomosis or reverse saphenous vein graft. Since patients with vascular injury experienced damage to the posterior structures, we performed simultaneous primary repair of extraarticular structures through the same skin incision immediately after vascular surgery. Supposedly, the exposure provided during vascular surgery provides us with a good surgical field and facilitates the repair of damaged tissue.

Acute surgery is usually defined as operative management performed within 3 weeks after injury, and it is advocated for the treatment of MLKIs before scar formation and tissue retraction [8, 11]. If possible, we performed primary repair as early as possible (within 1 week) because the damaged tissue is easy to identify. One disadvantage of acute repair is postoperative contracture, especially extension deficit, which is difficult to treat. Henley et al. evaluated patient and surgical factors that may potentially contribute to joint contracture following surgery [14]. Based on their results, no significant differences were observed in age, body mass index, associated injuries, or surgical timing. KDs and surgical intervention (on three or more ligaments) were associated with postoperative stiffness. In this series, most patients could achieve full knee extension. Since posterior structures of the knee become tight in knee extension, these structures should not be fixed in the flexion position. Supposedly, this is the reason why no extension deficit was observed in our patients. It is important to check whether the knee is fully extended after primary repair.

In the treatment of acute KDs and MLKIs, correct diagnosis and optimal primary repair of extraarticular ligaments are crucial to successful management. Extraarticular ligaments should be repaired where possible in the acute phase in the treatment of KDs and MLKIs. This treatment strategy can reduce the frequency of subsequent reconstructive surgery. Evidently, it is necessary to examine long-term results, such as progression of osteoarthritis, after these two treatments.

### Limitations

We acknowledge some limitations to this study. The most significant limitation is the nonrandomized study design. To compare staged surgery and one-stage reconstruction, it would be necessary to perform a randomized control trial (RCT). Since MLKIs consist of a small cohort with heterogeneous patient populations, accurate RCTs, as in ACL reconstruction, would be quite difficult to conduct. That primary repair of extraarticular structures improves outcome after cruciate ligament reconstruction in KD or MLKI can only be explained in a comparative study with a control group of patients who did not undergo primary repair. The same is true for the proposed reduction requiring cruciate ligament reconstruction. Furthermore, it is difficult to perform multiligament reconstruction, including the extraarticular ligaments, because we cannot obtain allografts in our country. The sample size was not large enough to show the effectiveness of the primary repair. However, the clinical outcomes were similar to those reported by Bin and Nam [18]. To achieve 80% statistical power with an *α* of 0.05 in demonstrating a large effect size (*r* = 0.5), power analysis revealed that a minimum of 53 patients in each group would be required for detecting any differences in clinical outcomes between the repair and staged groups using the Mann–Whitney *U* test. Therefore, further multicenter studies are needed.

## Conclusions

We retrospectively compared the outcomes between acute primary repair of extraarticular ligaments and staged surgery in KD and MLKI. All patients returned to their activities of daily living and preinjury occupation levels. Approximately 40% of the patients did not require further surgery and were just as satisfied with their surgical results as the staged group. Acute primary repair of extraarticular ligaments provides essential knee stability without varus/valgus instability and can decrease the need for subsequent cruciate ligament reconstruction.

## Data Availability

Not applicable.

## Abbreviations

KDs: Acute knee dislocations
MLKIs: Multiligament knee injuries
ROM: Range of motion
KOOS: Knee Injury and Osteoarthritis and Outcome Score
ACL: Anterior cruciate ligament
PCL: Posterior cruciate ligament
PMC: Posteromedial corner
PLC: Posterolateral corner
MCL: Medial collateral ligament
LCL: Lateral collateral ligament
CT: Computed tomography
